# Hearing and vision health for people with dementia in residential long term care: Knowledge, attitudes and practice in England, South Korea, India, Greece, Indonesia and Australia

**DOI:** 10.1002/gps.5563

**Published:** 2021-05-05

**Authors:** Piers Dawes, Iracema Leroi, Nisha Chauhan, Woojae Han, Vijaykumar Harbishettar, Dona M.P. Jayakody, Louise Jones, Adamos Konstantinou, Asri Maharani, Angelita Martini, Antonios Politis, Suhan Prabhakar, Sandra Prew, Costis Prouskas, Gregor Russell, Angus Sturrock, Sri Sunarti, Joanne Taylor, Theofanis Vorvolakos, Mark Worthington

**Affiliations:** ^1^ Department of Linguistics Macquarie University Sydney Australia; ^2^ Manchester Centre for Audiology and Deafness University of Manchester Manchester UK; ^3^ Global Brain Health Institute Trinity College Dublin Dublin Ireland; ^4^ Division of Neuroscience and Experimental Psychology University of Manchester Manchester UK; ^5^ Greater Manchester Mental Health NHS Foundation Trust Manchester UK; ^6^ Division of Speech Pathology and Audiology Hallym University Chuncheon Republic of Korea; ^7^ Nightingales Medical Trust Bengaluru Karnataka India; ^8^ Ear Science Institute Perth Australia; ^9^ Northumbria Healthcare NHS Foundation Trust North Tyneside UK; ^10^ Department of Psychiatry Larissa University General Hospital Faculty of Medicine University of Thessaly Larisa Greece; ^11^ Division of Nursing, Midwifery and Social Work University of Manchester Manchester UK; ^12^ Brightwater Research Centre Brightwater Care Group Perth Australia; ^13^ Department of Psychiatry National and Kapodistrian University of Athens Eginition Hospital Athens Greece; ^14^ Department of Psychiatry Division of Geriatric Psychiatry and Neuropsychiatry John’s Hopkins Medical School Baltimore USA; ^15^ NIHR Clinical Research Network (CRN) West Midlands Birmingham UK; ^16^ Aktios Care Home Units Athens Greece; ^17^ Bradford District Care NHS Foundation Trust Bradford UK; ^18^ Bradford District Care NHS Foundation Trust Bradford UK; ^19^ Division of Geriatric and Medical Gerontology Department of Internal Medicine Medical Faculty Brawijaya University Malang Indonesia; ^20^ Solent NHS Trust Southampton UK; ^21^ Department of Psychiatry Alexandroupolis University General Hospital Faculty of Medicine, Democritus University of Thrace Alexandroupolis Greece; ^22^ Lancashire & South Cumbria NHS Foundation Trust Sceptre Point, Sceptre Way Preston UK

**Keywords:** dementia, KAPsurvey, LTC, nursing home, shearing care, staff training, vision care

## Abstract

**Objectives:**

Up to 90% of people with dementia in long term care (LTC) have hearing and/or vision impairment. Hearing/vision difficulties are frequently under‐recognised or incompletely managed. The impacts of hearing/vision impairment include more rapid cognitive decline, behavioural disturbances, reduced quality of life, and greater care burden. This research investigated LTC staff knowledge, attitudes and practice regarding hearing/vision care needs for residents with dementia.

**Methods:**

A survey of staff in LTC facilities in England, South Korea, India, Greece, Indonesia and Australia. Respondents used a five‐point scale to indicate agreement or YES/NO response to questions regarding sensory‐cognitive care knowledge (what is known); attitudes (what is thought); practice (what is done).

**Results:**

Respondents reported high awareness of hearing/vision care needs, although awareness of how to identify hearing/vison difficulties or refer for assessment was low. Most felt that residents were not able to use hearing/vision devices effectively due to poor fit, being poorly tolerated or lost or broken devices. A substantial minority of respondents reported low confidence in supporting use of assistive hearing/vision devices, with lack of training the main reason. Most staff did not undertake routine checking of hearing/vision devices, and it was rare for facilities to have designated staff responsible for sensory needs. Variation among countries was not significant after accounting for staff experience and having received dementia training.

**Conclusions:**

There is a need to improve sensory support for people with dementia in LTC facilities internationally. Practice guidelines and training to enhance sensory‐cognitive knowledge, attitudes and practice in professional care teams is called for.

## Key points

- A survey of long term care (LTC) staff in six countries care revealed similar limitations in capacity to support residents with hearing/vision needs
- Better capacity to support hearing/vision needs of people with dementia was associated with longer experience working in LTC and having received training in dementia awareness
- Practice guidelines, training and fostering a long‐term professional work force could optimise support for hearing/vision needs for people living in LTC

## 1 INTRODUCTION

Up to 75% of people in long term care (LTC) have dementia.^1^ Up to 90% have hearing impairment (>25 dB HL)^2^ and >50% have visual impairment (visual acuity <6/12).^3^ Hearing/vision impairments among people with dementia are associated with reduced quality of life and increased agitation, hallucinations, aggression and depression,^4^ social isolation^5^ cognitive decline^6^ and higher care need.^7^ ^,^ ^8^ Identifying and treating hearing/vision impairment may improve outcomes for people with dementia.^9^ Unfortunately, hearing/vision impairments are under‐identified and under‐treated in people in LTC.^3^ ^,^ ^10^ Systematic reviews identified limitations on hearing/vision care in LTC including lack of staff knowledge, under‐use of screening tools, poor management of assistive aids (i.e., hearing aids, glasses, lighting) and the complex needs of residents with dementia.^11^, ^12^, ^13^

Previously, we undertook an exploration of knowledge, attitude and practice in relation to hearing/vision support for people with dementia in 117 English LTC facilities.^14^ Although staff recognised the impact of hearing/vision impairment, they lacked knowledge, skills and training to support hearing/vision needs. Considering global demographic shifts and rising need for LTC worldwide,^15^ we extended our investigation to include five additional countries, representing high, middle and lower socioeconomic strata with different experiences and numbers of LTC. We purposely included high income countries with long‐established LTC systems (England and Australia), countries that recently established a system of institutional LTC (South Korea), and countries where institutional LTC remains rare (Greece, India, Indonesia). The research questions were (i) What are the knowledge, attitudes and practice of LTC staff regarding sensory‐cognitive health?; (ii) are there differences in knowledge, attitudes and practice across countries?; and (iii) Does level of formal education, length of employment in LTC or training in dementia awareness relate to knowledge, attitudes and practice?

## 2 METHODS

### 2.1 Study design and population

A cross‐sectional multi‐national survey of staff who work with residents with dementia to investigate self‐reported knowledge, attitudes and approaches to hearing/vision care. We focused on LTC settings including residential homes which provide accommodation, meals and personal care and ‘nursing homes’ which employ nurses to support those with complex health needs.

*England*: Most LCT is user‐funded. Only those with assets below means‐tested levels receive publicly funded care. Residential care homes and nursing homes include those owned and run by local government, not‐for‐profit facilities owned by charities and for‐profit facilities.

*South Korea*: Korea introduced means‐tested publicly subsidised LTC in 2008 in response to population ageing, low income security for older people, reduced availability of informal carers, smaller family sizes and changes in attitudes to care of older people away from family and towards state support. LTC provider numbers expanded from 2600 to 5000 between 2009 and 2016. Most (70%–80%) are small residential (<30) or group homes (<10 residents), privately run and concentrated in urban areas.

*India*: LTC remains mostly home‐based with family members providing care. However, changing socioeconomic and cultural norms are driving increased demand for institutional LTC. In 2007, India enacted legislation making relatives of older people responsible for their maintenance and welfare, and requiring local governments to set up LTC for those without family support. LTC is unregulated and there is no national system of financial support. Because no LTC license is needed, the number of care homes is unknown. Most private sector LTC services are financially out of reach of most people.

*Indonesia*: Most LTC is provided by family, with assistance from community‐based volunteer centres. But changing social dynamics including smaller family sizes and younger people moving to cities makes care of older people a growing challenge. Institutional LTC is rare, and includes government‐run facilities for people who do not have families or financial ability to support themselves. The cost of private sector services is prohibitive for most people.

*Greece*: LTC remains primarily the responsibility of the family. LTC services fall short of demand and are of limited coverage, particularly outside urban centres. LTC for people without family or financial means is provided by publicly funded social care units or residential/nursing homes. Most homes are run by for‐profit organisations, the rest run by the Church and local authorities. For‐profit providers focus on dementia care. Publicly funded services for people with dementia are mostly unavailable. Professional carer roles are not formally recognised, with no minimum standards of training or service provision.

*Australia*: Access to publicly subsidised LTC is based on means‐tested assessment of income and assets. The level of government subsidy is based on individual assessment of care needs. LTC providers include not‐for‐profit, for‐profit and government providers across residential and nursing home services, with not‐for‐profit organisations being the largest provider (56%).

*Respondents*: We invited LTC staff who provide care for residents with dementia, including (i) Nurses and allied health professionals, including occupational therapists, physiotherapists and other trained health care professionals; or (ii) Paid non‐professional care workers, including front line care workers and others who assist residents with activities of daily living. We aimed to sample at least 50% of staff at each facility.^16^

### 2.2 Data collection

England: we used the Care Quality Commission (CQC) list to identify the 1274 care homes in Yorkshire and Humber, West Midlands and North East regions that provide care for older people with dementia. We randomly selected 17 care homes; 12 agreed to participate (71% response rate). South Korea: 20 care homes were randomly selected from lists of facilities in Seoul and Gangwon province; 14 agreed to participate (70% response rate). India: The survey was advertised in three residential dementia care centres in Karnataka state. Responses were received from all three centres (100% response rate). Indonesia: A database of facilities was used to identify seven care homes in Malang Regency; five agreed to participate (71% response rate). Greece: We approached three LTC facilities including two residential homes in Thessaly and Evros and a nursing home for people with dementia in Attica; all participated (100% response rate). Australia: The survey was advertised for one month in all 12 facilities in one not‐for‐profit organisation. Responses were received from all 12 facilities (100% response rate). A local member of the research team visited the participating facilities to provide information about the study. Managers of participating LTC facilities were asked to approach staff to complete the survey in paper, email, or online questionnaire format. Consent to participate was implied by completion of the survey. Ethical approval for the study was obtained from the London‐Surrey Borders Research Ethics Committee (England), Hallym University's Institutional Review Broad (HIRB‐2018‐065; South Korea), Nightingale Trust Institutional Review Board (India), Medical Faculty, University of Brawijaya Ethics Committee (232/EC/KEPK/08/2019; Indonesia), Eginition Hospital Research Ethics Committee, National and Kapodistrian University of Athens (ΩΖ5Ε46Ψ8Ν2‐7Τ4/2018 (Greece) and the University of Western Australia's Human Research Ethics Committee (RA/4/20/5433; Australia). Data collection took place between November 2018 and June 2019.

### 2.3 Measures

We recorded size, funding (public/local authority, private or not‐for‐profit) level of care (residential, nursing home, dementia specialist). ‘Public/local authority’ refers to homes that are taxpayer funded and government run. ‘Private’ refers to profit‐making enterprises. ‘Not‐for‐profit’ refers to homes run by charities including social co‐operatives and churches.

*Knowledge*, *attitudes and practice questionnaire* (Supplementary Appendix 1): A draft survey was collated from previous surveys of staff, residents with dementia and family/friends in relation to vision/hearing impairment in LTC.^3^ ^,^ ^10^ We consulted people with lived experience of cognitive, hearing/vision impairment to co‐produce the final survey. The survey was piloted by a small number of LTC professionals in the UK and adjusted according to their feedback. The survey was translated following forward and back translation methodology^17^ and adapted (e.g., with respect to local terminology) by collaborators at each site and piloted for comprehensibility and readability with local LTC professionals. Respondents were asked to respond to YES/NO statements or level of agreement (strongly disagree to strongly agree) on a five‐point Likert scale. Questions were clustered according to: Knowledge (what is known); attitudes (what is thought); practice (what is done), regarding hearing/vision care.

### 2.4 Statistical analysis

Responses were transferred to SPSS (version 25; IBM, Armonk, NY). LTC facility metrics, respondent demographics and survey responses were summarised descriptively. To test the validity and reliability of the knowledge, attitudes and practice survey and support quantitative analysis (in logits, the Rasch unit of measurement), data were analysed using the Polytomous Rasch Measurement model. RUMM2030Plus^19^ was used to carry out Rasch analysis on the combined data from all respondents (Supplementary Appendix). Linear multivariable regression was used to model variation in knowledge, attitudes and practice according to country, level of formal education, staff experience (i.e., length of employment in LTC) and training in dementia.

## 3 RESULTS

**FIGURE 1:**
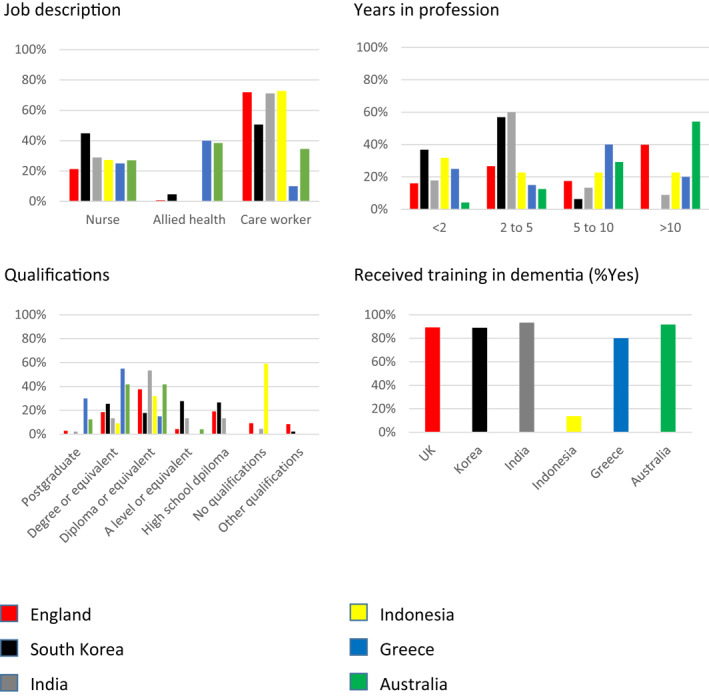
Demographics of survey respondents across England, South Korea, India, Indonesia, Greece and Australia

Responses were received from 428 workers in six countries (Table 1). Respondents were mostly female. Around 40% of respondents in Greece and Australia were allied health professional workers (Figure 1). In the remaining countries, respondents were either nurses or care workers. Australian respondents were relatively experienced, while Korean and Indian respondents were relatively inexperienced. Australian and Greek respondents were relatively highly qualified (following the higher proportion of allied health workers in those samples), while Korea and Indonesia had a relatively high proportion of respondents with basic levels of education. Most (80%‐83%) reported receiving training in dementia awareness. Only 14% of Indonesian respondents reported dementia training.

**TABLE 1:** Characteristics of the survey sites and the participating care homes and respondent numbers

Country	No. of care homes	Residents	Funding	Type	Respondents
England	11	28–117	Private 10 Local authority 1	Residential: 4 nursing: 5 dementia specialist: 2	*N* = 144
Australia	12	21–137	Not‐for‐profit 12	Residential: 0 nursing: 11 dementia specialist: 1	*N* = 24
Greece	3	94–289	Private 3	Residential: 2 nursing: 0 dementia specialist: 1	*N* = 20
South Korea	14	25–250	Private 14	Residential: 10 nursing: 0 dementia specialist: 4	*N* = 174
India	3	21–86	Not‐for‐profit 3	Residential: 0 nursing: 0 dementia specialist: 3	*N* = 44
Indonesia	5	10–34	Private 5	Residential: 4 nursing: 1 dementia specialist: 0	*N* = 22

### 3.1 Knowledge

Apart from Korean and Indonesian respondents, most reported knowing which residents have a hearing/vision impairment and use hearing aids, glasses or other aids (Figure 2). Most respondents reported being aware of hearing/vision screening tests, but not how to administer or interpret them. The majority of respondents in Korea, Australia and England reported not being aware of referral pathways and a substantial minority (majority of Koreans) reported not knowing how to incorporate hearing/vision needs in management plans. Korean respondents and a substantial minority of those from other countries reported not being confident in helping residents with use of assistive hearing/vision aids, mainly due to lack of training.

FIGURE 2Knowledge of care home workers in relation to identification and management of hearing and vision impairment in people with dementia living in long term care (LTC) facilities

**Figure:**
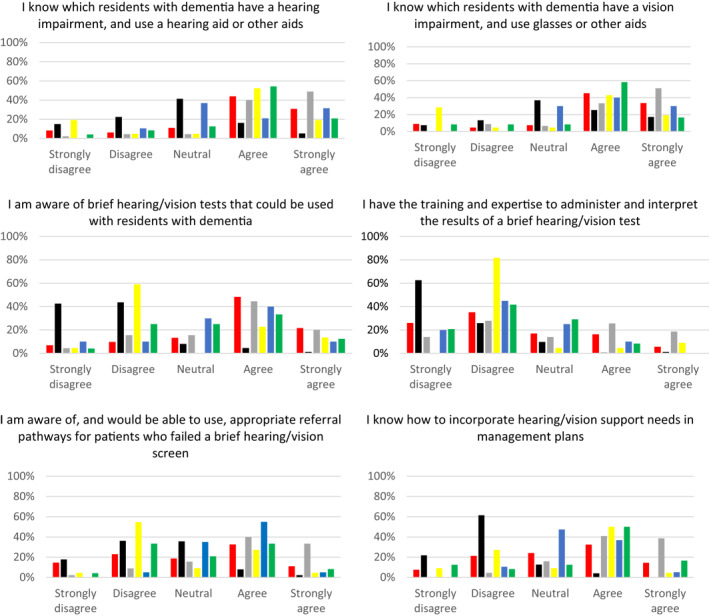

**Figure:**
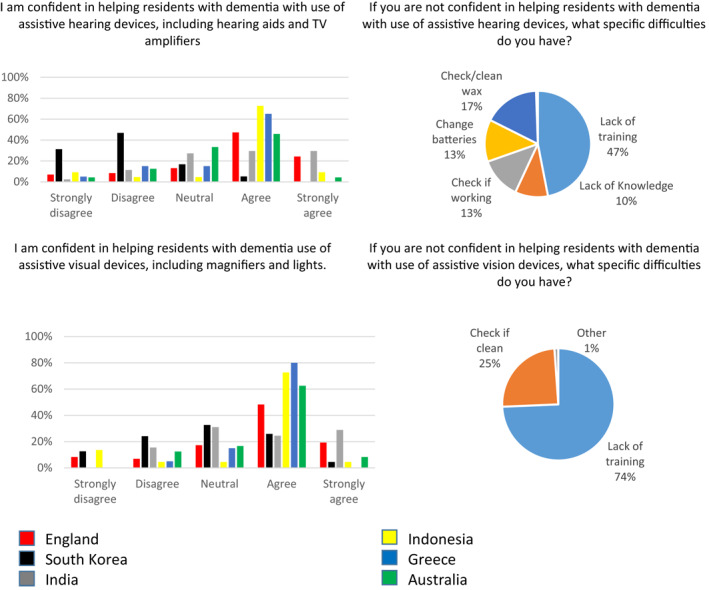

### 3.2 Attitude

**FIGURE 3:**
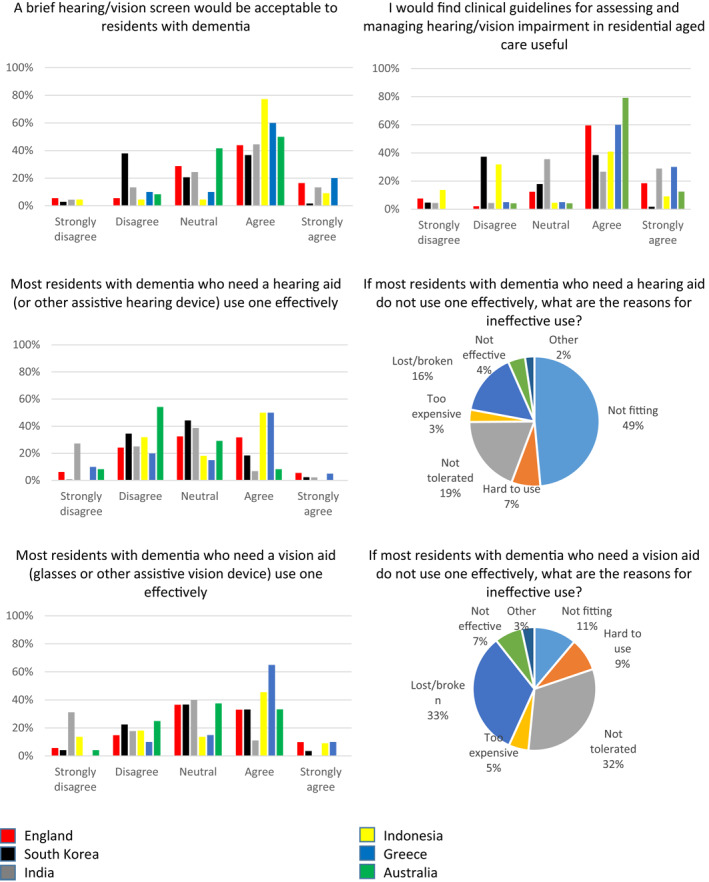
Attitudes of care home workers in relation to identification and management of hearing and vision impairment in people with dementia living in long term care (LTC) facilities

Korean respondents were ambivalent, but most respondents agreed hearing/vision screening would be acceptable to residents and would find clinical guidelines for assessment and management of hearing/vision useful (Figure 3). Apart from Greek respondents, most reported that residents who needed to use a hearing/vision aid did mostly not use them effectively. The most commonly reported reasons for ineffective use were aids not fitting, not being tolerated or being lost/broken.

### 3.3 Practice

**FIGURE 4:**
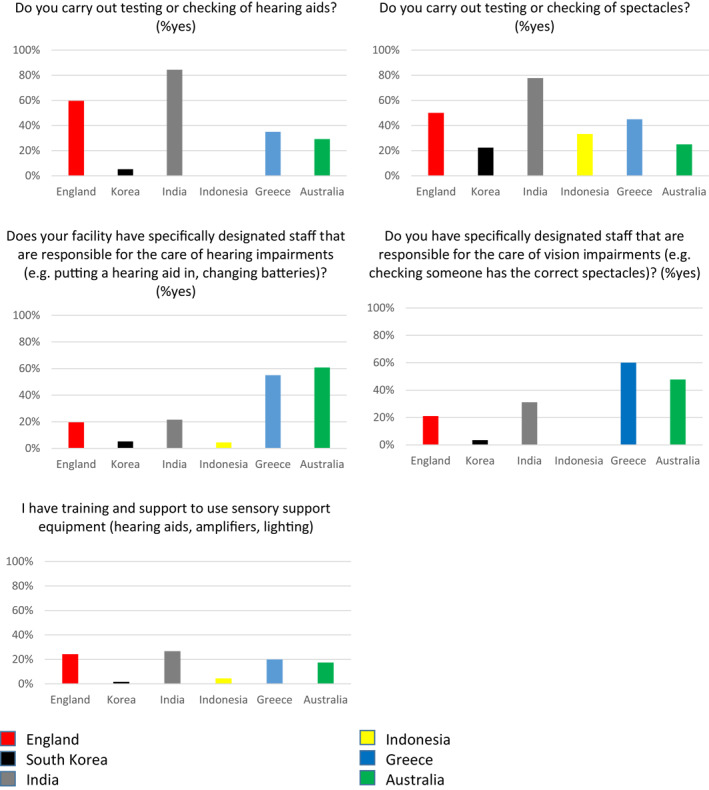
Practice of care home workers in relation to identification and management of hearing and vision impairment in people with dementia living in long term care (LTC) facilities

Most English and Indian respondents reported carrying out testing or checking of hearing aids and spectacles (Figure 4). Routine checking was reported to be lower elsewhere, particularly in Korea and Indonesia. 50%–60% of Australian and Greek respondents reported there were specially designated staff responsible for hearing/vision care. Most respondents elsewhere reported no specially designated staff. Most (75%–98%) staff reported not having training and support to use sensory equipment.

### 3.4 Multivariable analysis

Mean ‘capacity’ scores derived from Rasch analysis suggested variability between countries in capacity to identify and manage hearing/vision impairments in people with dementia in LTC (Supplementary Appendix). Australian, Indonesian and Korean respondents were most pessimistic about their capacity. In a multivariable regression model accounting for differences in staff education, training and experience, country and level of formal education were not significant correlates of capacity. Only staff experience (OR 0.20, 95% confidence interval 0.14–0.26) and having received training in dementia awareness (OR 0.25, 95% confidence interval 0.06–0.44) were associated with capacity, accounting for 11% of variation in capacity (*p* < 0.001).

## 4 DISCUSSION

Across six countries, LTC staff reported low capacity to support residents with dementia with hearing/vision needs. The six countries included those with established institutional LTC (England and Australia), recently established LTC (Korea) and countries where institutional LTC remains rare (Greece, India and Indonesia). Apart from those in Korea and Indonesia, most staff reporting knowing which residents had a hearing/vision problem, but not knowing how to administer or interpret hearing/vision screening tests. Previous studies suggest that staff have mis‐placed confidence in identifying those with hearing/vision needs without formal screening.^3^ ^,^ ^10^ Most staff reported not being confident in supporting use of hearing/vision assistive devices due to not having training or support. There was variability between countries in practice, though most staff reported not doing routine checks of hearing/vision assistive devices or having a specially designated person responsible for hearing/vision care.

Korean respondents tended to be particularly pessimistic about their capacity to support hearing/vision needs. Korea does not have hearing/vision care training requirements for LTC staff and relatively low training standards and staffing levels.^20^ Working in a LTC in Korea is perceived as being undesirable due to the demanding nature of the work and low pay. These issues are not specific to Korea, however.^21^ The pessimism of Korean respondents may be due to a combination of a relatively inexperienced workforce and low levels of formal qualifications (compared with India, the other country in the present study with a relatively inexperienced workforce).

In the present study, most staff reported that most residents were not able to use hearing/vision devices effectively due to devices not fitting, not tolerated or being lost/broken. These findings accord with inadequate hearing/vision support in LTC remarked upon since the 1980s.^22^ ^,^ ^23^ In some countries, hearing/vision needs are assessed on admission to LTC. But assessment is reliant of self‐reported status or observations of the person carrying out the admission and these assessments under‐identify hearing/vision impairment.^24^ Hearing/vision needs may go unmet as needs go unrecognised.

LTC staff and hearing/vision clinicians may lack the skills and confidence to work with people with dementia,^3^ ^,^ ^25^ or assume that people with dementia would not benefit from hearing/vision intervention. But systematic reviews identified benefits in terms of increased quality of life, improved communication, improved functional ability and reduced behavioural and psychological symptoms of dementia following hearing/vision interventions for people with dementia.^9^ There have been efforts to develop hearing/vision support appropriate for the needs of residents in LTC settings,^26^ but these have not been widely implemented. The persistent inadequacy of support for hearing/vision needs in LTC facilities requires solutions that are appropriate for the setting and cater for the individual needs of residents.^27^ ^,^ ^28^ Implementation of effective hearing/vision support is challenging due to low levels of funding for LTC, high care needs of residents and time pressure on staff leading to prioritisation of needs that may be more visible and perceived to be more urgent. The workforce also presents a challenge for effective hearing/vision support due to generally low levels of formal training and high staff turnover.^29^ Findings from this study suggest there is a relationship between an inexperienced workforce and capacity to support hearing/vision needs. The present study also suggested that staff would welcome training and guidelines to provide hearing/vision care for residents. Staff experience and training in dementia awareness were associated with greater capacity to support hearing/vision needs, but these two factors only accounted for 11% of variance in capacity. One might expect that specific training in hearing/vision care may be a much stronger correlate of capacity.

The main limitation of the present study is study samples that may not be typical of LTC in each country, particularly for India, Greece and Australia where sampling was pragmatic rather than random. Relatively few responses were received for the Greek and Australian sites relative to the size of the respective LTC facilities. Low response rates for these countries may indicate data for these sites may be less representative than that of other countries. However, the facilities in the present study reflect the LTC paradigm in each country. One might also argue that because institutional LTC is rare in some countries in this study (Greece, India, Indonesia), the issues in relation to support for hearing/vision needs identified in this study are not reflective of the experience of most dependent older people who continue to be cared for by family members in traditional ways. One might predict that capacity to support hearing/vision needs may be even lower among traditional care settings, where care is provided by family members with no training and limited support for hearing/vision care needs of older people. But some countries have made a recent transition away from traditional care by family towards institutional care (Korea) and other countries (India, Greece, Indonesia) are experiencing pressure for greater reliance on institutional care due to social and demographic changes. Therefore, it is useful to understand capacity for hearing/vision support in those countries where institutional care is growing in importance as well as countries where institutional care is more typical.

Identification and support for hearing/vision needs among residents of LTC facilities is a long‐standing issue complicated by systemic challenges. The present study offers cause for optimism. Although there was variation in capacity to support hearing/vision needs across countries, the experience of the workforce and having had dementia training accounted for this variation. Hearing/vision support for people with dementia in LTC could be optimised by providing guidelines and training in hearing/vision support, providing dementia awareness training and fostering a long‐term professional work force.

## CONFLICTS OF INTEREST

None.

## Supporting information

Supplementary Material

Supplementary Material

Supplementary Material

## Data Availability

Anonymised data are available by request to the corresponding author.
