# Supplementary material for: Hearing and vision health for people with dementia in residential long term care: Knowledge, attitudes and practice in England, South Korea, India, Greece, Indonesia and Australia
Source: Int J Geriatr Psychiatry. 2021 May 5;36(10):1531–40. doi: 10.1002/gps.5563 (PMC8518517; doi:10.1002/gps.5563)
Supplement: Supplementary file 1 — Supplementary Material [file GPS-36-1531-s002.docx]

**Version: Nurses and allied health professionals**

**Demographic information:**

1. Gender:

| 1 | Male |  |
| --- | --- | --- |
| 2 | Female |  |

1. Ethnic or cultural background:

| 1 | White |  |
| --- | --- | --- |
| 2 | Mixed/multiple ethnic groups |  |
| 3 | Asian/Asian British |  |
| 4 | Black/African/Caribbean/Black British |  |
| 5 | Other ethnic group |  |

1. Job title:

| 1 | Service manager or deputy service manager |  |
| --- | --- | --- |
| 2 | Registered nurse (please state type: RGN/RMN/Other) |  |
| 3 | Care worker (care assistant, support worker, nursing home assistant) |  |
| 4 | Allied health professional (state type) |  |
| 5 | Other (state type) |  |

1. Years in profession:

| 1 | Less than 2 |  |
| --- | --- | --- |
| 2 | 2-5 |  |
| 3 | 5-10 |  |
| 4 | More than 10 |  |

1. Qualifications (please tick your highest qualification):

| 1 | Postgraduate qualification (master’s or doctoral degree) |  |
| --- | --- | --- |
| 2 | Degree or equivalent |  |
| 3 | Diploma, certificate or equivalent |  |
| 4 | A-level or equivalent |  |
| 5 | GCSE grades A*-C or equivalent |  |
| 6 | No qualifications |  |
| 7 | Other qualification |  |

1. Have you received training on dementia**?**

| 1 | Yes |  |
| --- | --- | --- |
| 2 | No |  |

**Knowledge (what is known)**

| *Regarding* ***RESIDENTS WITH DEMENTIA IN RESIDENTIAL AGED CARE,*** *please indicate how much you agree or disagree with each of the following statements by ticking the box that best describes how you feel (NB there are no right or wrong answers).*  1. I know which residents with dementia having a hearing impairment, and use a hearing aid or other aids.   \| 1 \| Strongly disagree \|  \| \| --- \| --- \| --- \| \| 2 \| Disagree \|  \| \| 3 \| Neutral \|  \| \| 4 \| Agree \|  \| \| 5 \| Strongly agree \|  \|   2. I know which residents with dementia have a vision impairment, and use glasses or other aids.   \| 1 \| Strongly disagree \|  \| \| --- \| --- \| --- \| \| 2 \| Disagree \|  \| \| 3 \| Neutral \|  \| \| 4 \| Agree \|  \| \| 5 \| Strongly agree \|  \|   3. I am aware of brief hearing/vision tests that could be used with residents with dementia.   \| 1 \| Strongly disagree \|  \| \| --- \| --- \| --- \| \| 2 \| Disagree \|  \| \| 3 \| Neutral \|  \| \| 4 \| Agree \|  \| \| 5 \| Strongly agree \|  \|   4. I have the training and expertise to administer and interpret the results of a brief hearing/vision test.   \| 1 \| Strongly disagree \|  \| \| --- \| --- \| --- \| \| 2 \| Disagree \|  \| \| 3 \| Neutral \|  \| \| 4 \| Agree \|  \| \| 5 \| Strongly agree \|  \|   5. I am aware of, and would be able to use, appropriate referral pathways for patients who failed a brief hearing/vision screen.   \| 1 \| Strongly disagree \|  \| \| --- \| --- \| --- \| \| 2 \| Disagree \|  \| \| 3 \| Neutral \|  \| \| 4 \| Agree \|  \| \| 5 \| Strongly agree \|  \|   6. I know how to incorporate hearing/vision support needs in management plans.   \| 1 \| Strongly disagree \|  \| \| --- \| --- \| --- \| \| 2 \| Disagree \|  \| \| 3 \| Neutral \|  \| \| 4 \| Agree \|  \| \| 5 \| Strongly agree \|  \|   7. I am confident in helping residents with dementia with use of assistive hearing devices, including hearing aids and TV amplifiers.   \| 1 \| Strongly disagree \|  \| \| --- \| --- \| --- \| \| 2 \| Disagree \|  \| \| 3 \| Neutral \|  \| \| 4 \| Agree \|  \| \| 5 \| Strongly agree \|  \|   a. If you are not confident in helping residents with dementia with use of assistive hearing devices, what specific difficulties do you have?   \| 1 \| Lack of training on use/maintenance \|  \| \| --- \| --- \| --- \| \| 2 \| Lack of knowledge regarding turning hearing aid on/off \|  \| \| 3 \| Checking to see if working \|  \| \| 4 \| Changing batteries \|  \| \| 5 \| Checking/cleaning for wax \|  \| \| 6 \| Other (state) \|  \|   8. I am confident in helping residents with dementia use of assistive visual devices, including magnifiers and lights.   \| 1 \| Strongly disagree \|  \| \| --- \| --- \| --- \| \| 2 \| Disagree \|  \| \| 3 \| Neutral \|  \| \| 4 \| Agree \|  \| \| 5 \| Strongly agree \|  \|   a. If you are not confident in helping residents with dementia with use of assistive vision devices, what specific difficulties do you have?   \| 1 \| Lack of training on use/maintenance \|  \| \| --- \| --- \| --- \| \| 2 \| Check to see if working \|  \| \| 3 \| Other (state) \|  \| |
| --- | --- | --- | --- | --- | --- | --- | --- | --- | --- | --- | --- | --- | --- | --- | --- | --- | --- | --- | --- | --- | --- | --- | --- | --- | --- | --- | --- | --- | --- | --- | --- | --- | --- | --- | --- | --- | --- | --- | --- | --- | --- | --- | --- | --- | --- | --- | --- | --- | --- | --- | --- | --- | --- | --- | --- | --- | --- | --- | --- | --- | --- | --- | --- | --- | --- | --- | --- | --- | --- | --- | --- | --- | --- | --- | --- | --- | --- | --- | --- | --- | --- | --- | --- | --- | --- | --- | --- | --- | --- | --- | --- | --- | --- | --- | --- | --- | --- | --- | --- | --- | --- | --- | --- | --- | --- | --- | --- | --- | --- | --- | --- | --- | --- | --- | --- | --- | --- | --- | --- | --- | --- | --- | --- | --- | --- | --- | --- | --- | --- | --- | --- | --- | --- | --- | --- | --- | --- | --- | --- | --- | --- | --- | --- | --- | --- | --- | --- |

| Comments |
| --- |

**Attitudes (what is thought)**

*Regarding* ***RESIDENTS WITH DEMENTIA IN RESIDENTIAL AGED CARE,*** *please indicate how much you agree or disagree with each of the following statements by ticking the box that best describes how you feel (NB there are no right or wrong answers).*

1. A brief hearing/vision screen would be acceptable to residents with dementia.

| 1 | Strongly disagree |  |
| --- | --- | --- |
| 2 | Disagree |  |
| 3 | Neutral |  |
| 4 | Agree |  |
| 5 | Strongly agree |  |

2. I would find clinical guidelines for assessing and managing hearing/vision impairment in residential aged care useful.

| 1 | Strongly disagree |  |
| --- | --- | --- |
| 2 | Disagree |  |
| 3 | Neutral |  |
| 4 | Agree |  |
| 5 | Strongly agree |  |

3. Most residents with dementia who need a hearing aid (or other assistive hearing device) use one effectively.

| 1 | Strongly disagree |  |
| --- | --- | --- |
| 2 | Disagree |  |
| 3 | Neutral |  |
| 4 | Agree |  |
| 5 | Strongly agree |  |

a. If most residents with dementia who need a hearing aid do not use one effectively, what are the reasons for ineffective use?

| 1 | Not fitting |  |
| --- | --- | --- |
| 2 | Hard to use |  |
| 3 | Not tolerated |  |
| 4 | Too expensive |  |
| 5 | Lost/or broken |  |
| 6 | Not effective |  |
| 7 | Other (state) |  |

4. Most residents with dementia who need a vision aid (glasses or other assistive vision device) use one effectively.

| 1 | Strongly disagree |  |
| --- | --- | --- |
| 2 | Disagree |  |
| 3 | Neutral |  |
| 4 | Agree |  |
| 5 | Strongly agree |  |

a. If most residents with dementia who need a vision aid do not use one effectively, what are the reasons for ineffective use?

| 1 | Not fitting |  |
| --- | --- | --- |
| 2 | Hard to use |  |
| 3 | Not tolerated |  |
| 4 | Too expensive |  |
| 5 | Lost/or broken |  |
| 6 | Not effective |  |
| 7 | Other (state) |  |

| Comments |
| --- |

**Practice (what is done)**

*Regarding the care of* ***RESIDENTS WITH DEMENTIA IN RESIDENTIAL AGED CARE****, please indicate YES or NO to each of the following statements (NB there are no right or wrong answers).*

1. Do you carry out testing or checking of hearing aids?

| 1 | Yes |  |
| --- | --- | --- |
| 2 | No |  |

If yes, how do you do this?

| 1 | Patient report |  |
| --- | --- | --- |
| 2 | Carer report |  |
| 3 | Checking whether it is working or not |  |

2. Do you carry out testing or checking of spectacles?

| 1 | Yes |  |
| --- | --- | --- |
| 2 | No |  |

If yes, how do you do this?

| 1 | Patient report |  |
| --- | --- | --- |
| 2 | Carer report |  |
| 3 | Checking whether it is working or not |  |
| 4 | Checking whether the person has the correct spectacles for near or distance viewing |  |

3. Do you have specifically designated staff that are responsible for the care of hearing impairments (e.g. putting a hearing aid in, changing batteries)?

| 1 | Yes |  |
| --- | --- | --- |
| 2 | No |  |

4. Do you have specifically designated staff that are responsible for the care of vision impairments (e.g. checking someone has the correct spectacles)?

| 1 | Yes |  |
| --- | --- | --- |
| 2 | No |  |

5. I have training and support to use sensory support equipment (hearing aids, amplifiers, lighting).

| 1 | Yes |  |
| --- | --- | --- |
| 2 | No |  |

| Comments |
| --- |

**THANK YOU VERY MUCH FOR COMPLETING THIS QUESTIONNAIRE**
