# Supplementary material for: Hearing and vision health for people with dementia in residential long term care: Knowledge, attitudes and practice in England, South Korea, India, Greece, Indonesia and Australia
Source: Int J Geriatr Psychiatry. 2021 May 5;36(10):1531–40. doi: 10.1002/gps.5563 (PMC8518517; doi:10.1002/gps.5563)
Supplement: Supplementary file 2 — Supplementary Material [file GPS-36-1531-s001.docx]

**Appendix 2**

The difference between total raw scores and between levels of ordinal survey items (e.g. Likert scale) is unknown, limiting potential to compare across respondents and precluding use of parametric statistical analyses. However, if data from a set of items can be shown to fit the RPM, that set of items represents a single variable and the person measures (in logits, the Rasch unit of measurement) from such an analysis form a linear scale that can be used in standard statistical procedures. The Rasch model transforms the probability of responses (e.g. degree on Likert scale) as a function of person and item parameters and has the properties of invariant or fundamental measurement typical of measurements in the physical sciences [1].

Because of the large discrepancies in sample sizes between the countries, the properties of the combined three scales were examined using only the data from the UK. The set of item locations from that analysis were then anchored and used as the item locations for the full data. Rasch analysis was carried out using the RUMM2030Plus software which offers a range of facilities to examine various aspects of validity and reliability for a set of items. The results for the most pertinent aspects are briefly described here – first for the UK sample and then for the full sample. The psychometric properties for the combined K, A and P scales for the UK data were judged to be sufficiently robust to proceed to the full sample analysis.

*Establishing the psychometric properties of the K, A and P items using the UK data*

Figure 1 shows the items and persons are well-targetted to each other. The Person Separation Index (equivalent of Cronbach’s Alpha) was 0.836, indicating good reliability.


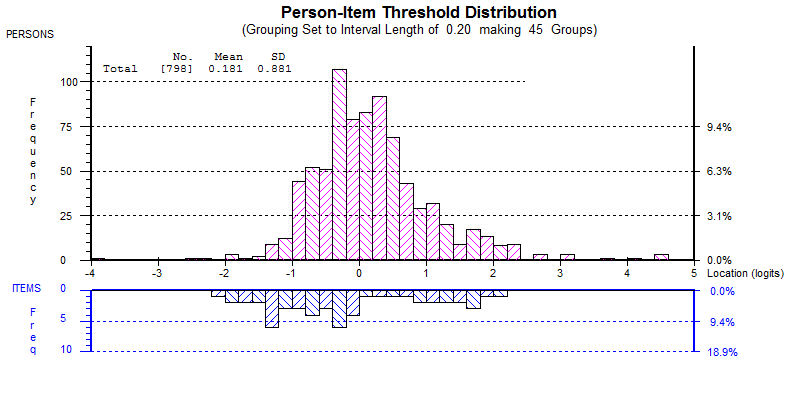


Figure 1. Distribution graph for person and Item threshold locations (the cut-points between successive response categories). Persons are at the top and items at the bottom of the graph.

Table 1. Item locations and fit statistics in increasing order of difficulty/intensity

| Item | Location | SE | FitResid | DF | ChiSq | DF | Probability* |
| --- | --- | --- | --- | --- | --- | --- | --- |
| K2 | -0.811 | 0.041 | -4.006 | 740.49 | 15.675 | 3 | 0.001323 |
| K1 | -0.788 | 0.041 | -3.434 | 739.55 | 14.907 | 3 | 0.001898 |
| K7 | -0.699 | 0.042 | -4.241 | 736.74 | 20.769 | 3 | 0.000118 |
| A2 | -0.599 | 0.047 | -0.447 | 734.87 | 1.745 | 3 | 0.627055 |
| K8 | -0.572 | 0.040 | 1.684 | 730.19 | 4.259 | 3 | 0.234833 |
| P1 | -0.497 | 0.079 | -0.037 | 729.25 | 8.315 | 3 | 0.039935 |
| A1 | -0.482 | 0.041 | 1.978 | 732.06 | 10.520 | 3 | 0.014626 |
| K3 | -0.454 | 0.039 | -1.678 | 735.81 | 11.404 | 3 | 0.009734 |
| A4 | -0.231 | 0.040 | 3.843 | 733.00 | 8.614 | 3 | 0.034884 |
| P2 | -0.153 | 0.077 | 0.690 | 725.51 | 8.577 | 3 | 0.035472 |
| K6 | -0.142 | 0.036 | -0.652 | 732.06 | 4.260 | 3 | 0.234701 |
| A3 | -0.019 | 0.039 | 6.302 | 737.68 | 26.506 | 3 | 0.000008 |
| K5 | 0.050 | 0.035 | 1.261 | 733.00 | 0.083 | 3 | 0.993751 |
| K4 | 0.580 | 0.037 | 3.465 | 729.25 | 19.544 | 3 | 0.000212 |
| P5 | 1.207 | 0.087 | 0.239 | 704.91 | 5.080 | 3 | 0.166025 |
| P3 | 1.764 | 0.098 | 2.607 | 709.59 | 5.266 | 3 | 0.153304 |
| P4 | 1.844 | 0.100 | 1.842 | 703.04 | 3.029 | 3 | 0.387115 |

- *Bonferroni adjusted p<0.00059*
- *Sample size adjusted statistically ( n=500) due to impact of sample size on chi sq.*

Three items showed some statistical misfit to the model – one over-discriminated (K7) and two under-discriminated (A3 and K4) relative to the rest of the items. However, when the Item Characteristic Curves (ICCs – showing theoretical (the curves) and observed (dots) expected values) for the two least well-fitting items are examined (see Figure 2), it may be seen that the misfit is small: in general, person locations increased across the range. (Note: fit here is referring to an ideal; not to some null hypothesis). On this basis, fit to the model is accepted as very good overall.


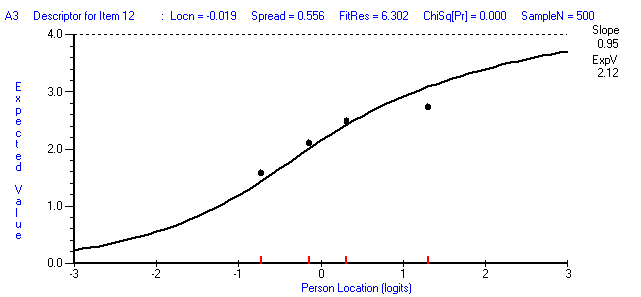

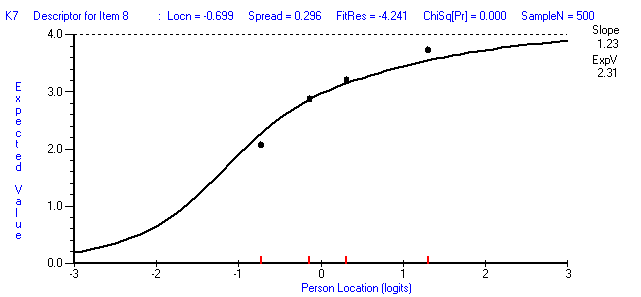


Figure 2. The two least well-fitting items, K7 (relative over-discrimination) and A3 (relative under-discrimination).

Some pairs of items showed response dependence, suggesting one of each pair can be considered redundant, eg K1/K2, P1/P2. Nevertheless, to ensure content validity, they need to be retained. Principal component analysis suggested the presence of two subscales after the common single scale is accounted for: one consisting of mostly K items and the other comprised of P and A items. At 1% level of probability, about 4.4% of persons may be better represented by measures on these two subscales.

*Analysis of full sample (all countries) using the item locations from the UK data.*

Figure 3 shows the items and persons to be well-aligned to each other: a very few persons at the highest (right) and lowest (left) ends of the person continuum are not being measured quite as reliably as all other persons. The Person Separation Index (reliability) was 0.861, indicating good reliability.


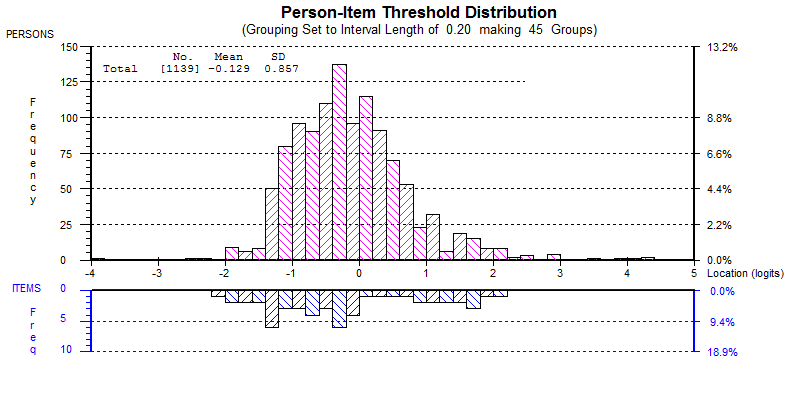


Figure 3. Distribution graph of person and item threshold locations for the full sample.

The three scales K, A and P can be equated to one another as shown in Figure 4. For example, a location of 1 logit corresponds to raw scores of about 2.5 on P, and about 12.5 and 23 on A and K .

Although K, A and P items are spread across the range of difficulty of item locations, P tends to be the more difficult to endorse overall. This pattern is evident also in Table 1.


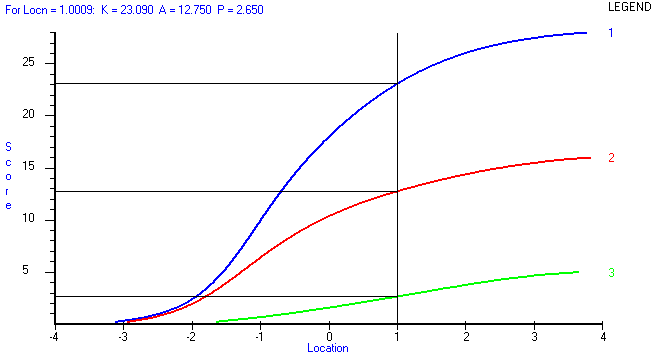


Figure 4. Equating K, A and P scale raw scores against logit locations.

No meaningful Differential Item Functioning analysis could be carried out for these data due to the small samples sizes for a number of countries.

Person Locations (in logits) from this analysis were used to carry out Multiple Regression analysis as described in the main text.

**References**

1. Andrich, D. and I. Marais, *A Course in Rasch Measurement Theory.* Measuring in the Educational, Social and Health Sciences. Singapore: Springer, 2019.
