# Supplementary material for: Hearing and vision health for people with dementia in residential long term care: Knowledge, attitudes and practice in England, South Korea, India, Greece, Indonesia and Australia
Source: Int J Geriatr Psychiatry. 2021 May 5;36(10):1531–40. doi: 10.1002/gps.5563 (PMC8518517; doi:10.1002/gps.5563)
Supplement: Supplementary file 3 — Supplementary Material [file GPS-36-1531-s003.docx]

Appendix

Country

Capacity (RUMM location score)

**Figure.** Mean capacity scores for England, Korea, India, Indonesia, Greece and Australia in relation to care home staff’s self-reported knowledge, attitude and practice in relation to identification and management of hearing/vision impairment in people with dementia living in long-term aged care. Error bars represent standard error of the mean.
